# Uptake of a primary care atrial fibrillation screening program (AF-SMART): a realist evaluation of implementation in metropolitan and rural general practice

**DOI:** 10.1186/s12875-019-1058-9

**Published:** 2019-12-06

**Authors:** Jessica Orchard, Jialin Li, Robyn Gallagher, Ben Freedman, Nicole Lowres, Lis Neubeck

**Affiliations:** 1 Heart Research Institute/Charles Perkins Centre, University of Sydney, Sydney, NSW 2006 Australia; 2 Susan Wakil School of Nursing, Faculty of Medicine and Health/Charles Perkins Centre, University of Sydney, Sydney, Australia; 3 Edinburgh Napier University, Edinburgh, UK

**Keywords:** Atrial fibrillation, Screening, Realist evaluation, General practice, Primary care

## Abstract

**Background:**

Screening for atrial fibrillation (AF) in people aged ≥65 years is recommended by international guidelines. The Atrial Fibrillation Screen, Management And guideline-Recommended Therapy (AF-SMART) studies of opportunistic AF screening in 16 metropolitan and rural general practices were conducted from November 2016–June 2019. These studies trialled custom-designed eHealth tools to support all stages of AF screening in general practice.

**Methods:**

A realist evaluation of the AF-SMART studies, which aimed to explain the circumstances in which the program worked (or not) to increase the proportion of people screened for AF. The initial program theory was based on our previous research, policy documents and screening studies. To test this, we conducted 45 semi-structured interviews with general practitioners (GPs), nurses and practice managers across all participating practices, and collected observational and quantitative screening data. These data were analysed and interpreted to refine the program theory.

**Results:**

GPs/nurses liked the eHealth tools, although technical problems sometimes disrupted screening. Time was the main barrier to screening for GPs/nurses, so systems need to be very efficient. Practices with leadership from a senior GP ‘screening champion’ had broader uptake, especially from the nursing team. Providing regular feedback on screening data was beneficial for quality improvement and motivation. Clear protocols for follow-up of abnormal results were required for successful nurse-led screening in a hierarchical system. Participation in the program had broader benefits of improving AF knowledge and raising the profile of cardiovascular health in the practice. Screening for a shorter, more intense period (eg during influenza vaccination) worked well for practices where sufficient staff time was allocated.

**Conclusions:**

Introducing an AF screening program is likely to be successful in contexts where there is a senior GP ‘screening champion’, a clear protocol exists for abnormal results, and there is regular data reporting to staff. These contexts link to mechanisms around motivation, leadership, empowerment of nurses, and efficient screening systems. The contexts and mechanisms contribute to the longer-term outcomes of increasing the proportion of people screened and treated for AF, which is recommended by guidelines as a key strategy for the prevention of AF-related stroke.

**Trial registrations:**

AF SMART (metropolitan): ACTRN12616000850471 (Australia New Zealand Clinical Trials Registry).

AF SMART II (rural): ACTRN12618000004268 (Australia New Zealand Clinical Trials Registry).

## Background

### Gaps in AF screening and treatment

Atrial fibrillation (AF) is the most common heart arrhythmia. The prevalence of AF increases with age, and if untreated, raises the risk of stroke fivefold [1]. AF is commonly asymptomatic, and approximately 1.4% of people aged ≥65 years have undiagnosed AF [2]. AF-related stroke is highly preventable with appropriate oral anticoagulant (OAC) treatment. For those at high risk of stroke (with a CHA_2_DS_2_-VA [3] risk score ≥ 2), OAC treatment can reduce the risk of stroke by 64% [4].

A number of guidelines and expert consensus statements now recommend opportunistic single-timepoint AF screening for people aged ≥65 years [1, 3, 5]. These guidelines generally advocate screening by pulse palpation or single-lead electrocardiogram (ECG). Despite these recommendations, screening is not routinely performed opportunistically in practice. A survey of general practitioners (GPs) published in *The Economist* found that respondents in Australia had only screened 11% of eligible patients in the previous fortnight [6].

Historically, there were substantial gaps in treatment for patients diagnosed with AF. Treatment rates were previously around 50–60% [7] but are reported to be increasing in England, Spain and Denmark [8–10]. These increases are largely due to the introduction of non-vitamin K dependent OAC (NOAC) drugs.

### The AF-SMART studies

The Atrial Fibrillation Screen, Management And guideline Recommended Therapy (AF-SMART) studies of opportunistic AF screening implementation in metropolitan [11] and rural [12] general practice were conducted by our research group from November 2016–June 2019.

These studies had very similar methods, which have been described previously [11, 12]. Briefly, a convenience sample of 16 general practices (8 metropolitan and 8 rural) in New South Wales, Australia were recruited. Each practice was provided with several smartphone handheld single lead ECGs (iECG) (Kardia Mobile, Alivecor [13]), together with several custom-designed eHealth tools to support all stages of screening. These tools were:

1. **A screening prompt**: this app was located on a third-party hosting platform and used information extracted in real-time from the electronic medical record to show a small screening prompt when an eligible patient’s file was opened. Eligible patients were those attending the practice who were aged ≥65 years with no previous AF diagnosis and who had not been screened with the iECG in the past 12 months. The visibility of the prompt was improved during the study based on user feedback. Practices also recorded the iECG automated result for each screening in this app.
2. **Electronic Decision Support (EDS)**: the EDS app was also located on the same third-party platform and guided evidence-based treatment for those diagnosed with AF. The EDS used information from the patient’s electronic medical record to calculate the patient’s CHA_2_DS_2_-VA score [3] and associated stroke risk, and made OAC treatment recommendations based on guidelines.
3. **Data reporting for Quality Improvement (QI)**: de-identified, custom-designed data extracts were obtained every 1–2 months from practices. Practices were then provided with regular QI reports of their AF screening data based on these data extracts. Information in these reports included the total number screened by each GP/nurse, the number of normal/abnormal results, the percentage of eligible patients screened, the number of confirmed cases of AF and the proportion of these treated according to guidelines. The level of detail provided in the QI reports was increased in the rural practices, in order to attract specific QI continuing professional development (CPD) points for GPs.

Practices conducted screening for a median of 5 months (range: 2–12 months). Practice nurses and GPs were both able to screen patients. All follow-up of patients with abnormal screening results, including other diagnostic tests, management and whether specialist referral was required, was at the discretion of the treating GP. Some practices chose to undertake screening for shorter, more intense periods, e.g. during influenza (flu) vaccination clinics. Practices were reimbursed a small amount for screening (about AU$10 per patient screened, plus $1000 to cover IT costs and setup time) to mimic potential “real world” Medicare funding.

Key study outcomes included: the proportion of eligible people screened for AF, and the proportion of people with AF treated according to guidelines.

### Realist evaluation

The realist evaluation framework seeks to build a program theory to identify the reasons interventions affect changes differently in different contexts. It is a theory-driven and method neutral evaluation framework based on a series of informed hypotheses developed for scientific research by Pawson and Tilly [14]. Realist evaluation asks the key questions of ‘what works’, ‘for whom’ and ‘in what situation’. It analyses practices within the realm of interacting psychosocial and cultural systems, which consists of multiple actors, contexts, and mechanisms. Realist evaluation does not assume outcomes result directly from interventions, because the interaction between an intervention and an outcome can vary and produce a rippling effect that changes the outcomes at other levels of the system.

Central to realist evaluation is the context (C) + mechanism (M) = outcome (O) formula as a guiding principle for enquiry. This configuration links logical models of interventions (mechanisms) with circumstances (contexts), with the aim to demonstrate the intricacies of the interconnected causal relationships that resulted in desired or unintended changes (outcomes). Contexts are conditions that a specific intervention operates in. Mechanisms are ‘generative’ and refer to a combination of interacting stakeholder reasoning and resources, which triggers stakeholder responses that result in an outcome. Outcomes are effects resulting from the conditions created by mechanisms operating in a context and comprise intended and unintended effects of a program. Just as there are multiple actors, contexts and mechanisms in place, realist evaluation uses multiple outcome measures to analyse a program’s success.

Realist evaluation has been widely applied to public health and QI programs and healthcare interventions to explain outcome differences and to test and refine a program theory to determine how/why a program works. In particular, it has been used to assess complex healthcare programs aimed at behaviour changes and quality improvement, e.g. a UK quality improvement program to increase NOAC prescription for AF in general practice [15], an individual health intervention to reduce pelvic floor prolapse for women [16], a local-level intervention to improve paediatric and neonatal clinical practices [17], and in a large government program to support normal birth [18].

There are usually three phases in realist evaluation [18]. These are:

1. developing the initial program theory, which is tested and refined during the study;
2. testing the program theory through mixed methods of data collection; and
3. analysing data to refine the program theory and context-mechanism-outcome (CMO) configurations.

Realist evaluations often use multiple data sources. The overall aim is for the data to “allow analysis of CMOs relevant to the program theory and to the purposes of and the questions for the evaluation” [19]. A mixed-methods approach is often used, including both quantitative data (e.g. surveys or clinical audit data) and qualitative data (e.g. focus groups, semi-structured interviews) sources [20]. Evaluation of complex programs requires more thorough analysis of implementation contexts. The success of a program or intervention often depends on individual responses as well as the wider context [19], and interview data can allow researchers to provide a deeper level of understanding. For example, semi-structured interviews of GPs, nurses and practice managers provided detail about the dynamics of primary care practices, underlying financial or non-financial motivations, AF knowledge, and clinical staff views on patient preferences, providing a more nuanced understanding of the contexts in which the AF screening program was operating. The evaluation was conducted concurrently with the AF-SMART studies, from November 2016 – June 2019 and aimed to describe and explain the circumstances in which the AF screening program (including the custom designed eHealth tools) worked (or not) to increase the proportion of eligible people screened for AF.

## Methods

### Phase 1: developing the initial program theory

The initial program theory was developed using a number of sources. These sources included: a review of findings in previous studies of AF screening; discussions with stakeholders (GPs, practice nurses, researchers and cardiologists); and examination of data and findings from our previous AF screening pilot studies [21, 22].

### Phase 2: testing the program theory

The initial program theory was tested by collecting data during the AF-SMART studies. Primary modes of data collection were semi-structured interviews, observation and quantitative screening data.

#### Semi-structured interviews

Semi-structured interviews were conducted with 45 staff (21 GPs, 13 nurses and 11 practice managers) across each study site towards the end of the study period in that practice. A purposive sampling approach was used, and at each practice we sought to interview GPs and nurses who had participated in the screening program, ideally including some staff who had done small numbers and some who had done large numbers of screenings to obtain a range of views. Written informed consent was obtained from each subject prior to the interview.

Interviews explored participants’ views on the screening program, iECG device, electronic tools (the screening prompt and EDS), deidentified quantitative data collection and QI reporting. GPs and nurses were asked to discuss their views on various barriers and enablers to screening, including: confidence in performing screening, how long it took, their impression of whether patients liked the screening process, how abnormal results were dealt with and treatment of those diagnosed with AF (Additional file 1: Table S1). The interviews were audio recorded and transcribed verbatim.

#### Observation

We observed staff at practices during the AF-SMART studies. Observation followed an ethnographic approach [23] and occurred during practice visits and through phone calls and emails with practice staff, GPs and nurses. Key points for observation included barriers and enablers to screening, attitudes to the value of screening and the level of motivation to participate. Observational data were recorded in all practices (*n* = 8) using field notes and researcher reflective diaries and were analysed together with transcripts of semi-structured interviews using an inductive thematic framework approach, as described below. Our data from observations, together with the interview and quantitative data, assisted in creating a deeper understanding of the causal pathways linking the complex processes [23] involved in the screening program.

#### Quantitative screening data

Quantitative screening data were obtained every 1–2 months from each participating practice using a customised, deidentified clinical audit tool. Data included the number of screenings done by each GP/nurse, the iECG automated interpretation, details of AF diagnoses and treatment, most recent visit date to the practice and some demographic data. These data extracts were used to report back to practices about their study progress, and formed part of a QI clinical audit (eligible for CPD points) in the AF SMART rural study.

### Phase 3: refining the program theory

#### Semi-structured interviews

Semi-structured interview transcripts, together with observation field notes and researcher reflective diaries, were analysed using a thematic framework approach. This approach classifies data according to key themes, commonalities and patterns [24, 25]. An inductive process was used to construct the coding framework based on the four initial interviews, which was then used to systematically code the remaining interview transcripts by two members of the research team. Refinements and additions were made to the framework as required until thematic saturation was reached, i.e. when no new themes or codes emerged [26]. These data were iteratively reflected on, shared and discussed with the broader research team to refine the CMO configurations and key propositions.

#### Quantitative screening data

Screening data at each practice were analysed to show the number people screened during the study and the proportion of eligible patients screened who attended the practice during the study period. These data were then used to show the overall ‘success’ of each practice and were linked to refined CMO configurations and key propositions.

### Ethical approval

The AF-SMART studies had approval from the University of Sydney Human Research Ethics Committee (Protocol No. 2017/1017 and Protocol No. 2014/962).

This paper has been written to comply with the RAMESES II reporting standards for realist evaluation [19].

## Results

### Phase 1: initial program theory

Essentially, the findings of phase I of the evaluation were that many individual screening tools do not work very well in isolation and that a comprehensive and efficient system is needed. In relation to screening, our previous studies showed both GPs and nurses liked screening with the iECG device, but that there were several key barriers, including time pressure (both for screening and follow-up of abnormal results), and lack of funding/remuneration for screening [21, 22]. In relation to treatment of patients with AF, a range of interventions have been developed to increase effective prescribing of guideline-recommended anticoagulation in primary care settings. These include use of electronic decision support tools [27], targeted GP-education programs [28], nurse-led clinics [29], and patient-focused education interventions [30]. Overall, these interventions increase effective prescribing, but the results are varied and may decrease over time [30].

**Fig. 1:**
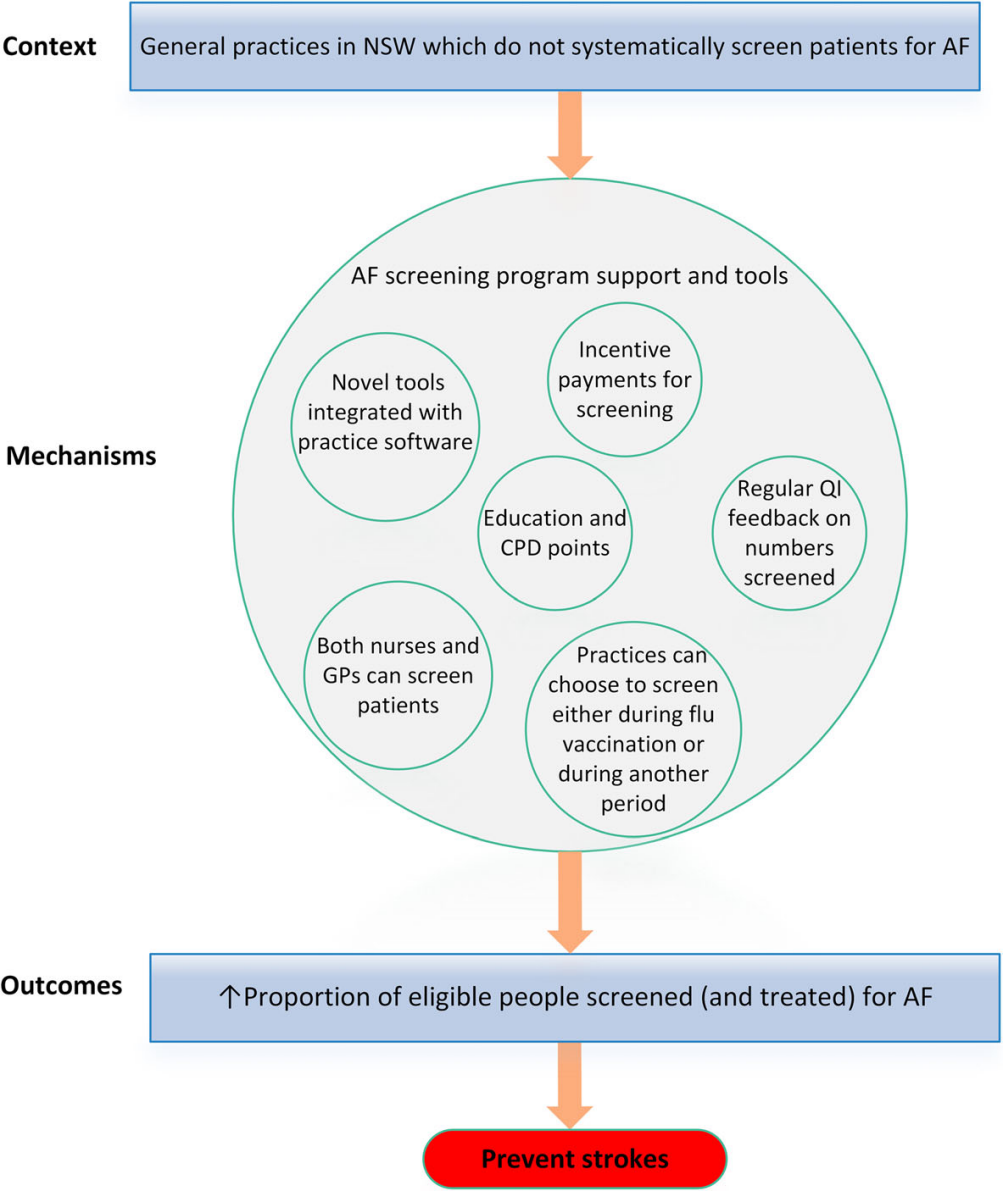
initial program theory. *AF* atrial fibrillation; *EDS* electronic decision support; *iECG* smartphone electrocardiograph; *CPD* continuing professional development; *QI* quality improvement

Therefore, our initial program theory was that by providing a novel screening device (the iECG) together with integrated electronic tools (including a screening prompt to automatically identify eligible patients) and financial incentives to support all stages of screening, the program will be more systematic and efficient, and therefore time-poor GPs and nurses will opportunistically screen a higher proportion of eligible patients aged ≥65 years seen in the practice (Fig. 1). Guidelines and expert consensus [1, 3, 5, 31] recommend increasing screening (and treatment), including in the primary care setting, to prevent more strokes.

### Phase 2: testing the program theory: key propositions

**Fig. 2:**
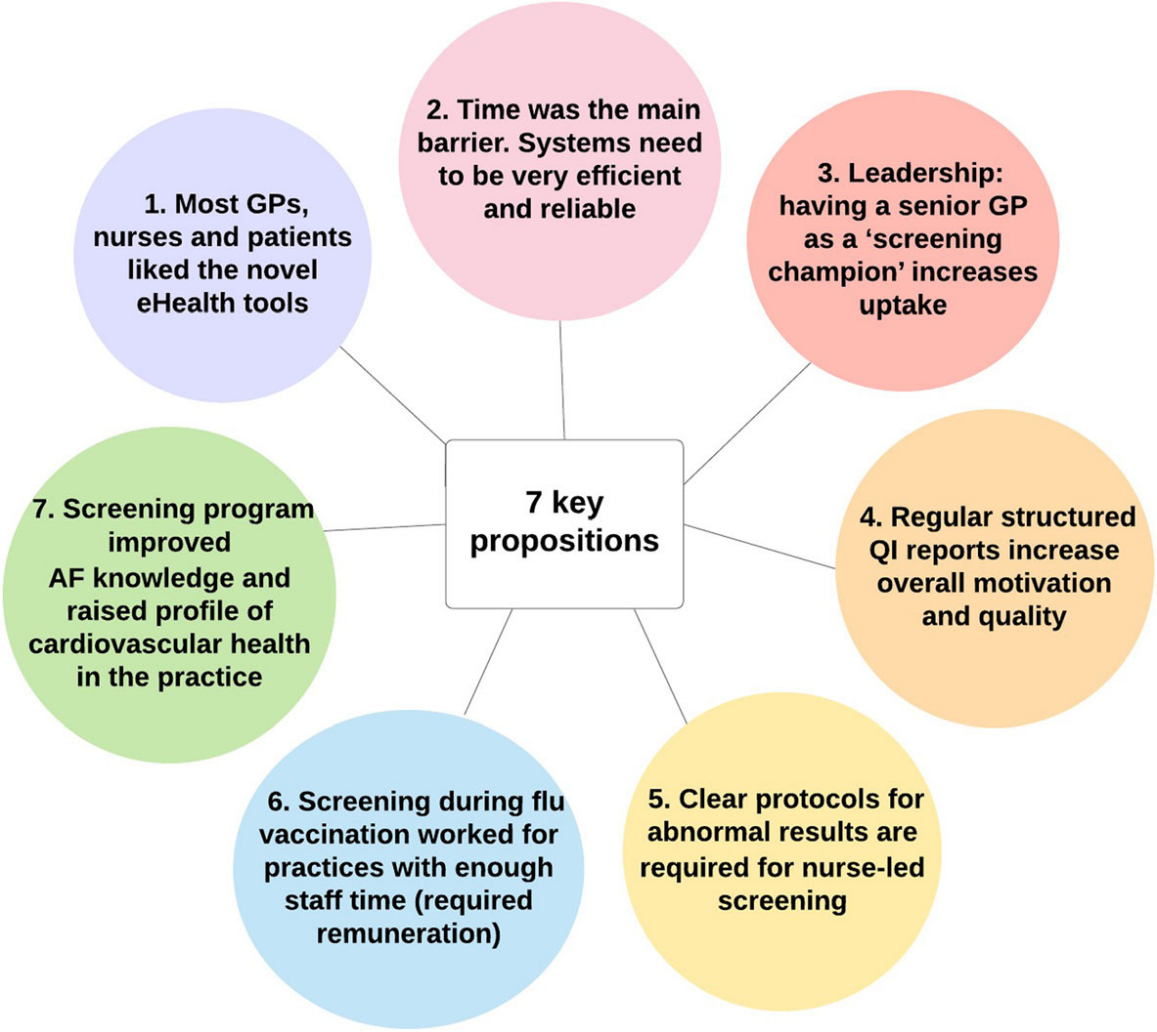
summary of 7 key propositions

Seven key propositions were identified based on key themes emerging from the data collected in phase 2 (Fig. 2):

#### Proposition 1: Most GPs, nurses and patients liked the novel eHealth tools, especially the iECG device and improved prompts

All GPs and nurses interviewed stated that they liked the iECG device. They also reported a high level of patient satisfaction with the device. The real-time trace on the smartphone screen together with the automated interpretation were engaging, and the device was cutting-edge, which participating GPs felt reflected well on the practice:

*“The patients really quite enjoyed it and I quite enjoyed playing with a little machine too. I quite enjoyed the technology side of it.”* (GP 1, Practice A).

*“Patient acceptability has been really high…it’s so easy and quick”* (GP 1, Practice C).

*“I think it’s very encouraging…to get an ECG rhythm strip and look at it immediately to see a regular heart rhythm.”* (GP 2, Practice C).

*“Patients absolutely loved it. Everyone loves new technology and…. It reflects well on me in a sense.”* (GP 1, Practice I).

All except one of the interviewees were very confident using the device and performing screening, partly due to the ubiquity of smartphones.

*“I felt confident. It was very easy to do.”* (Nurse, Practice A).

The only difficulty with the iECG was taking a reading for people with a tremor or arthritic fingers:

*“The only difficulty was with the elderly with tremors. They got to a certain age, probably about 85, where they just couldn’t…it was almost like a screen for cognitive status.”* (GP 2, Practice A).

*“Some of them had essential tremor…I just couldn’t get a tracing.”* (GP 1, Practice I).

A minority of interviewees (less than a quarter) reported resistance to technology, either in relation to their own attitudes or as a perceived barrier to uptake at their practice:

*“I’m probably technically challenged sometimes…I don’t want to push any wrong buttons.”* (Nurse, Practice P).

*“Some GPs are uncomfortable and uncertain with new technology.”* (GP 1, Practice I).

The improved prompt was a useful tool for nurses and GPs who participated in screening, and meant that they did not forget about screening:

*“The prompt was very useful…it reminded me to [screen].”* (GP 1, Practice A).

#### Proposition 2: Time was the main barrier to screening for GPs/nurses who have little control over their time. Systems need to be very efficient, and reliability is key with eHealth tools

More than three-quarters of respondents indicated that time was the main barrier to AF screening in general practice. This was the case even for GPs and nurses who were generally very motivated. Importantly, screening was always done as an ‘adjunct’ task – that is, it was not the main reason patients were at the practice – and therefore, it added time to the consultation. Beyond a certain point, performing screening risked ‘hijacking the consultation’:

*“It was something to be done on quieter days, not horrendous clinical days… when we were running behind time, you tended not to get the screening done because it was on top of what the consultation was about.”* (GP, Practice H).

GPs and particularly nurses both had relatively little control over their time:

*“The nurses’ schedule seems to be so hectic they did what they could…it’s more like a mini emergency room at times so it made it difficult.”* (Practice Manager, Practice P).

*“[Patients have] their own list of things that we’ve got to get through and then we’ve got a couple of things that we might need to do urgently, so it was often a struggle to find the time to do it.”* (GP 2, Practice I).

In this context, systems need to be very efficient and electronic tools need to work well, otherwise they add time. There were some reliability issues with the eHealth tools, which interrupted screening and undermined trust in the electronic tools.

*“When it works it’s great, and I love all the things. It’s just so unreliable…that unless you’ve got someone like me who really is willing to give it a go…[others] don’t have the time.”* (GP, Practice E).

*“[the prompt and EDS] wouldn’t work for quite a while, and then the phone wouldn’t work for some reason. It was fixed eventually.”* (GP 1, Practice A).

While the iECG device was quick to use, often extra time was required to explain the device, screening process and rationale to the patients took extra time, particularly compared to pulse palpation.

*“I could take a pulse for 30 seconds or I could spend 3 minutes explaining to them the device and where to put their fingers and telling them what we were going to do. That’s the difference…as far as time use.”* (Nurse, Practice H).

*“It took 5-10 minutes because it meant you did a lot more talking about preventative stuff, explaining things.”* (GP, Practice H).

#### Proposition 3: Leadership: having a senior GP as a ‘screening champion’ increases uptake across the practice especially from the nursing team

In most practices, a small number of GPs were very engaged with the program and screened a lot of people themselves. Usually, these were senior GPs (eg a partner in the practice), and when they provided this leadership, i.e. acting as a ‘screening champion’, there was broader uptake in screening across the practice, especially from the nursing team. The GP champion provided leadership and increased motivation, as well as reinforcing the idea of practice-level participation:

*“A couple of GPs really took it on, while a couple of them found it a burden. [A GP champion] spoke to them and said ‘we really need to be doing this’.”* (Nurse, Practice A).

Nurses also performed better when there was a GP champion as well as a nursing team leader providing leadership with screening:

*“We work together and we just say ‘oh that’s right we do that’, so usually we can work through [problems].”* (Nurse, Practice P).

*“I don’t feel motivated to do it because nobody else was interested.”* (Nurse, Practice N).

Interestingly, there were still a substantial number of GPs in each practice who either did not participate at all, or only to a very limited degree, in screening.

*“A few nurses and one of the doctors really ran with it…. we tend to find in a larger practice we get varied levels of engagement.”* (Practice Manager, Practice G).

#### Proposition 4: Regular structured screening data reports, including number screened and whether newly diagnosed patients are treated according to guideline, increase overall motivation and quality

Practices appreciated receiving regular screening data reports, including the number of people with AF, the number screened by each staff member and the proportion of AF patients treated according to guideline. In many cases, GPs had never seen their practice data presented in that way. In some cases, it led to quality improvement, e.g. review of management for AF patients who were not previously treated according to guideline.

*“From a practicing GP’s point of view, you need to keep an eye on these people [patients not eligible for OAC at the time of AF diagnosis] because as time goes by, they can drift into the category that probably does benefit from anticoagulant.”* (GP, Practice D).

*“When I saw the data…my first reaction was that’s actually a really good study.”* (Practice Manager, Practice I).

These data reports often increased motivation, and facilitated internal competition:

*“Feedback’s always great and timely feedback is particularly important. It is lovely to have been able to receive timely feedback each month, and we have enjoyed the friendly competition within our team!”* (GP 2, Practice C).

*“The nurses got really competitive because [the GP champion] kept telling them who’d done the most.”* (Practice Manager, Practice G).

*“Just on the reporting...We’re very competitive so I love the fact that I’m leading the nursing team. Our nurse who has just come back from maternity leave is keen to see the next round of data so she can see her name up there”* (Nurse, Practice C).

#### Proposition 5: Clear protocols for follow-up of abnormal results are required for nurse-led screening to be successful

Approximately 16–20% of iECG readings were abnormal (either unclassified or possible AF) and required at least some follow-up. Therefore, nursing teams performing screening need a clear and efficient protocol for dealing with abnormal screening results.

*“If we had worked it out with our GPs beforehand, and had a clear protocol of how to move forward, it would have saved us a bit of to-ing and fro-ing between the GPs and myself.”* (Nurse, Practice I).

This is particularly important in the context of a hierarchical general practice system, where nurses may not be empowered to take next steps for follow up without GP input.

*“A nurse can’t tell the patient anything. The doctor can at least provide some insight into what the result means.”* (Practice Manager, Practice I).

#### Proposition 6: screening for a shorter, more intense, period (eg during flu vaccination) worked well for practices with sufficient staff time allocated to screening. This required remuneration for screening

About one quarter of practices found that screening patients intensively for a shorter period worked well. Perhaps it is easier to maintain motivation and ‘momentum’ for a shorter period. For example, screening during dedicated flu clinics worked well in practices where there was extra time allocated for nursing staff and a clear protocol for dealing with abnormal results. Providing remuneration for screening in the context of a fee-for-service primary care system was important, as it provided an incentive to allocate extra staff time to screening.

*“Thankyou also for being one of the rare research studies to have been aware enough of the pressures on primary care to have built practice remuneration into your research protocol.”* (GP 1, Practice C).

The main benefit of screening during flu vaccination was that it captured a high proportion of eligible patients who do not otherwise attend the practice regularly.

*“I think it’s…rolling it in as a systematic process, and then that means we are doing it once a year for everybody.”* (GP, Practice E).

When these factors were absent, practices felt they would not have time to screen during flu vaccination:

*“The nurses were seeing one patient every 7 minutes to do the flu shot and therefore didn’t really get round to doing this.”* (Practice Manager, Practice K).

#### Proposition 7: Participating in an AF screening program had broader benefits of improving AF knowledge and raising the profile of cardiovascular health in the practice

More than half of the interviewees commented on how their knowledge of AF had improved as a result of participating in the study and that it had raised the profile of cardiovascular health in their practice.

*“It made me improve my ‘patter’ about AF. I got the statistics right in my head about risks per annum per patient and what we were doing to try and get them down, which helped my medicine.”* (GP 2, Practice A).

*“When I do a study and I’m focused on a particular topic, it does help me to be more diligent and vigilant on that topic.”* (GP 1, Practice C).

*“[Screening] is adding to our conversation about heart health”.* (Nurse, Practice C).

#### Phase 3: refined program theory

**Fig. 3:**
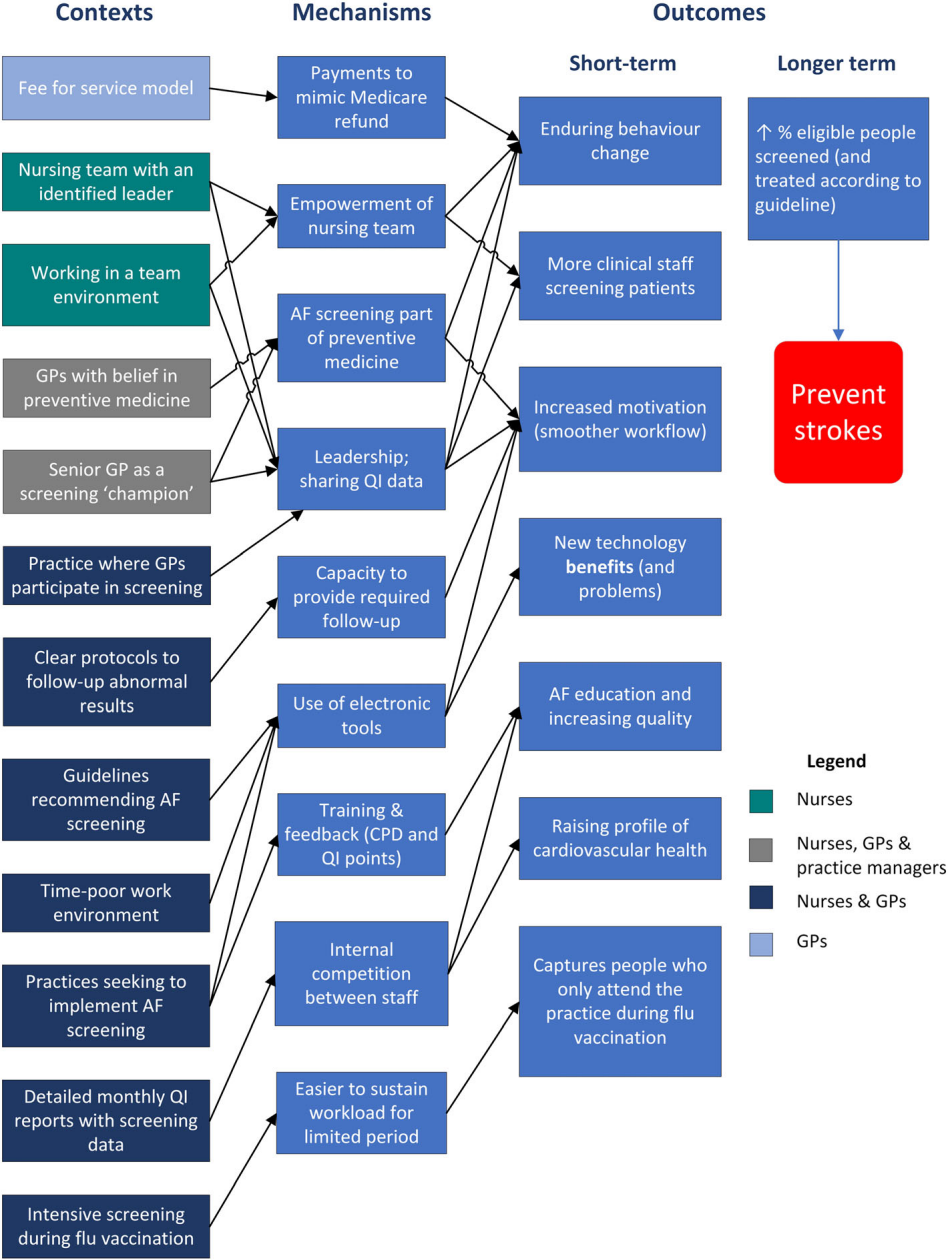
refined program theory. *AF* atrial fibrillation; *iECG* smartphone electrocardiograph; *CPD* continuing professional development; *QI* quality improvement

The refined program theory context, mechanism and outcome (CMO) configurations are presented in Fig. 3. Contexts included factors related to the regulatory environment of general practice in Australia, such as practice nurse remuneration. Nine mechanisms were identified, and 7 short term outcomes were identified. Short term outcomes primarily related to behaviour change, motivation, and barriers and enablers to screening. Longer term outcomes were increasing the proportion of eligible people screened (and treated according to guideline), and ultimately preventing strokes.

Individual practice screening data are presented in Table 1. This includes the number of patients screened and the proportion of eligible patients screened (across the whole practice, not just for GPs/nurses who participated in screening). In addition, links between these data and refined program theory CMO configurations are included. As can be seen, the 5 practices screening the highest numbers and proportion of eligible patients have GP champions, an engaged nursing team with a leader and well-established protocols for following-up abnormal results.

**Table 1:** Individual practice screening data and links to refined program theory

Site	Eligible patients screened (%)	Eligible patients screened (n)	Link to CMO configuration
Practice A (rural)	51%	611	- senior GP champions
			- nurse leadership
			- internal competition
			- detailed QI reporting & improved prompt
Practice B (rural)	48%	473	- senior GP champion
			- nurse-led, teamwork
			- detailed QI reporting & improved prompt
Practice C (rural)	43%	582	- senior GP champion
			- nurse leadership
			- internal competition between nurses
			- detailed QI reporting & improved prompt
Practice D (rural)	42%	696	- nurses screening intensively during flu vaccination clinic
			- detailed QI reporting & improved prompt
Practice E (metro)	33%	231	- senior GP champion
			- nurses screened intensively during GP-led flu vaccination clinic
Practice G (rural)	28%	445	- senior GP champion
			- internal competition between nurses
			- detailed QI reporting & improved prompt
Practice F (metro)	27%	322	- senior GP champion screening opportunistically
Practice H (rural)	19%	125	- GP and nurses screening
			- very small practice
			- detailed QI reporting & improved prompt
Practice I (metro)	19%	690	- senior GP champion
			- very large practice
			- dedicated preventive care nurses screening
Practice J (metro)	19%	263	- GP champion, nurse also screening opportunistically
Practice K (metro)	16%	120	- several GPs screening less intensively over longer period
Practice L (rural)	15%	69	- very small nursing team
			- detailed QI reporting/improved prompt
Practice M (rural)	9%	102	- only 1 GP screening within medium-size practice
Practice N (metro)	6%	59	- mainly single practice nurse screening
Practice O (metro)	5%	66	- nurses screening opportunistically over longer period
Practice P (metro)	4%	55	- mainly 1 GP screening within large practice
**TOTAL SCREENED**		**1806 (metro)** **3103 (rural)** **4909 (combined)**	

*CMO* context, mechanism, outcome, *Metro* metropolitan, *GP* general practitioner, *QI* quality improvement

## Discussion

### Summary of findings

This evaluation identified and explained the circumstances in which an opportunistic AF screening program in general practice worked to increase the proportion of eligible people screened for AF. To our knowledge, this is the first realist evaluation to systematically link specific individual and practice-level mechanisms of action with program outcomes in relation to AF screening.

The results suggest that key mechanisms for program success are practice-wide engagement driven by senior GP leadership and regular QI feedback, and high user acceptance of novel eHealth tools including an automated prompt to identify eligible patients. In some practices, undertaking intensive screening program over a shorter period (e.g. during annual flu vaccination) worked well, provided sufficient staff time was allocated and that there was a clear protocol for follow-up of abnormal results.

Our initial program theory focussed on eHealth resources providing the mechanisms, but our refined theory found that mechanisms were also highly related to internal motivation. Therefore, while providing novel and efficient electronic tools was a helpful resource for motivated GPs and nurses, a more nuanced approach may be needed to encourage a broad uptake, especially by GPs, in each practice.

### Comparison with existing literature

A number of previous studies have evaluated the feasibility of screening for AF using a single-lead rhythm strip to detect unknown AF in general practice [11, 21, 22, 32, 33]. Similar issues in terms of barriers and enablers to AF screening were also described in a pharmacy screening program [34]. As in this study, our previous pilot studies of AF screening in general practice also found that GPs and nurses really like the iECG device, and that nurses were very confident providing screening [21, 22]. Guidelines and expert consensus statements now expressly state that AF screening may be performed using an ECG rhythm strip (as an alternative to pulse palpation) [1, 5]. The increased specificity and sensitivity of digital tools [13, 35] compared with pulse palpation [36] are important advantages of new technology. It is suggested that this should be considered in future guideline updates, as improved screening accuracy may reduce the need for specialist review of the diagnosis, and therefore improve patient management, providing timely and appropriate initiation of thromboprophylaxis with oral anticoagulant. However, as the guidelines do recommend echocardiographic assessment, and selection of a rate- or rhythm-control strategy, this will generally require referral to a specialist.

This evaluation highlighted the importance of having a senior GP screening ‘champion’ together with a nursing team with a clear leader. While we previously noted the varied engagement in the screening process in the AF-SMART metropolitan study [11], it appeared engagement levels were generally higher in the rural practices. These practices almost all had a clear GP champion who was a senior member of the practice and a very engaged nursing team with clear leadership. This combination was highly influential for success. Previous studies have also emphasised the key role of an empowered ‘organisational change champion’ together with a ‘project champion’ in successfully implementing and sustaining quality improvement initiatives in primary care [37, 38].

Frequent QI reporting provided motivation for practices. The AF-SMART metropolitan study found that the clinical audit data could be further improved [11]. The rural practices also showed the benefits of more detailed, frequent QI reporting as part of an enhanced ‘audit and feedback’ system. These reports facilitated internal competition between GPs and nurses regarding screening numbers at several practices, as well as several examples where the treatment of newly diagnosed patients was reviewed based on the QI reporting. This is consistent with a 2003 Cochrane review which that found audit and feedback systems improve clinical performance [39], and also with a 2012 Cochrane review which found that the success of audit and feedback systems depends both on baseline performance and how feedback is provided [40].

Screening intensively for a shorter period of time (e.g. during flu vaccination) was an effective model in practices that were well prepared. This was similar to the results in our 2016 pilot study where nurses conducted AF screening during flu vaccination [22], which found that this method of screening could be very successful provided sufficient time was allocated and there was a clear protocol at the outset for follow up of abnormal results. AF screening during flu vaccination clinics has also been found to be feasible in the Netherlands [32] and is being piloted in the UK [41].

### Strengths and limitations

Realist evaluation is a practical approach for understanding the real-world program constraints, such as time and structural limitations. In terms of data collection, it was a strength that all interviews were conducted by the same researchers, and the sample was representative and appropriate in that staff from all practices participated in the interviews.

There were some limitations in the data collection. Our semi-structured interviews only captured a sample of each practice, and may not reflect the views of all GPs and nurses in that practice. However, significant bias is unlikely given the overall number of interviews conducted and that several staff (with varying levels of screening numbers) were interviewed from each practice. Also, we note that interviews can be potentially affected by participants’ desire to be socially acceptable and a reluctance to criticise the project [42]. However, we do not consider this would have been likely to affect the validity of the findings as the topic was not particularly emotive, interviews were framed as an opportunity to give feedback, the sampling strategy was diverse and the responses were relatively consistent.

### Practical implications

While our study findings are strongly context-related, it is possible they could translate to different primary care interventions within and/or to other health systems internationally. These findings could be used by policymakers designing and implementing screening or QI programs in general practice. In particular, our findings suggest that the identification of a senior GP ‘champion’ together with provision of audit and feedback data (including at the individual staff member level), may be useful mechanisms to support practice-wide motivation/engagement to implement various programs in general practice. The ubiquity of electronic medical records systems in general practice, together with clinical audit tools to efficiently extract and analyse data, will provide increasing scope for QI feedback in future programs. Training health professionals in the acquisition, interpretation and dissemination of QI data within the practice may play a key role in the future success of implementation programs in general practice.

## Conclusions

Our realist evaluation suggests that introducing an AF screening program is likely to be successful in contexts where there is a senior GP ‘screening champion’, a clear protocol exists for abnormal results, and there is regular data reporting to staff. These contexts link to mechanisms around motivation, leadership, empowerment of nurses, and efficient screening systems. Together the contexts and mechanisms raise the profile of cardiovascular health in the practice and contribute to the longer-term outcomes of increasing the proportion of people screened (and treated) for AF, which is recommended by guidelines as a key strategy for the prevention of AF-related stroke. Future programs need to specifically work on strategies to increase internal motivation and thereby encourage a broader uptake of screening in general practice.

## Supplementary information

**Additional file 1: Table S1.** Interview guide for semi-structured interviews.

## Data Availability

The full datasets generated and/or analysed during the current study are not publicly available as individual privacy may be compromised and data sharing is not permitted by our ethics approval. Some data are available from the corresponding author on reasonable request.

## Abbreviations

AF: Atrial fibrillation
AF-SMART studies: Atrial Fibrillation Screen, Management And Guideline Recommended Therapy studies
CHA_2_DS_2_-VA: C, congestive heart failure/left ventricular dysfunction; H, high blood pressure; A2, age older than 75 years; D, diabetes mellitus; S2, stroke/transient ischemic attack/thromboembolism; V, vascular disease (coronary artery disease, myocardial infarction, peripheral artery disease, aortic plaque); A, age 65 to 74 years
CMO: Context, mechanism, outcome
CPD: Continuing professional development
ECG: Electrocardiogram
EDS: Electronic decision support
Flu: Influenza
GP: General practitioner
iECG: Smartphone electrocardiogram
IT: Information technology
QI: Quality improvement
