# Supplementary material for: Uptake of a primary care atrial fibrillation screening program (AF-SMART): a realist evaluation of implementation in metropolitan and rural general practice
Source: BMC Fam Pract. 2019 Dec 6;20:170. doi: 10.1186/s12875-019-1058-9 (PMC6896363; doi:10.1186/s12875-019-1058-9)
Supplement: Supplementary file 1 — Additional file 1: Table S1. Interview guide for semi-structured interviews. [file 12875_2019_1058_MOESM1_ESM.docx]

Supplementary Table 1 – interview guide for semi-structured interviews

| **GPs** | **Practice nurses** | **Practice managers** |
| --- | --- | --- |
| *Screening program – views* | | |
| - How satisfied were you with the screening process overall? - What aspects of the screening program do you think were useful for your patients and your practice? - What did you like about the screening program? Did you find it useful and convenient? - What is your estimate of the average amount of time that AF screening added to the amount of time you usually spend per patient? - Did you read the QI reports? Were they useful? | - How satisfied were you with the screening process overall? - On a scale of 1 to 10, with 10 being very confident and 1 being not at all confident, how confident were you with doing the screening? - Do you feel you received sufficient training and support? If not, what extra training or support would have been useful for you? - Were there any things you liked about the screening process? Did you find it convenient? - What is your estimate of the average amount of time that AF screening added to the amount of time you usually spend per patient? - Did you read the QI reports? Were they useful? | - How satisfied were you with the screening program overall? - Do you feel the staff received sufficient training and ongoing support? If not, what extra training or support would have been useful for them? - Was there a cost to providing the screening to your practice? Do you think the financial assistance provided was sufficient to cover the cost? - In what ways did the screening program (including data collection) impact on the workflow of the practice? Do you have any comments on the amount of work involved for you? - Did your practice participate in any other studies during the AF screening program? |
| *Areas for improvement in the screening program* | | |
| - Was there anything you didn’t like or found difficult with the screening program? - Did the screening program interfere with workflow in the practice? - What are your recommendations for improving the screening process? | - Were there any aspects of the screening process that you did not like or were difficult for you? If so, can you describe these in more detail? - Do you have any recommendations for improving the screening process? | - Were there any aspects of the screening process that you did not like or were difficult for your practice? If so, can you describe these in more detail? |
| *Patient perspective* | | |
| - Did you feel it made you more engaged with your patients? - How did your patients respond to the screening program? | - What aspects of the screening program do you think were useful for patients? - How did you feel the patients responded to the screening? | - Did your patients find the AF screening valuable? |
| *Follow up and diagnosis* | | |
| - Did your practice have clear protocol for abnormal results? - How did your practice deal with unclassified results? - Did you do or order a 12 lead ECG on patients with positive result for AF? | - How did you communicate abnormal results to the GP and the patients? - Did you do or request a 12 lead ECG on patients with abnormal screening results? - Did you have any false positive results? If so, what was the impact? |  |
| *AF treatment / knowledge* | | |
| - Did you use the EDS? If the answer is yes, in what way, if any, did the EDS assist with your knowledge of atrial fibrillation-related stroke risk? - As a result of the study, did you change the management of any patient with known or new AF? Did you change management regarding OAC? | - Did you take part in the AF training for nurses? If yes, do you have any comments on the training? - Did the screening program and the nurses’ training (if attended) assist with your knowledge of atrial fibrillation and AF-related stroke risk? |  |
| *AF screening in future* | | |
| - Can you see a sustainable role of screening for AF outside of the flu/shingles vaccination period, and what would that be? | - Can you see a sustainable role of screening for AF outside of the flu/shingles vaccination period, and what would that be? | - Would your practice consider running an AF screening program again in future? |
| *Other issues* | | |
| - Are there any other issues not covered that you would like to talk about? | - Are there any other issues not covered that you would like to talk about? | - Are there any other issues not covered that you would like to talk about? |

AF, atrial fibrillation; GP, general practitioner; EDS, electronic decision support; OAC, oral anticoagulant; ECG, electrocardiogram
